# The Stability of Comorbid Psychiatric Disorders: A 7 Year Follow Up of Children with Pervasive Developmental Disorder-Not Otherwise Specified

**DOI:** 10.1007/s10803-015-2592-5

**Published:** 2015-10-12

**Authors:** C. Verheij, A. Louwerse, J. van der Ende, M. L. J. M. Eussen, A. R. Van Gool, F. Verheij, F. C. Verhulst, K. Greaves-Lord

**Affiliations:** Department of Child and Adolescent Psychiatry/Psychology, Erasmus Medical Center/Sophia Children’s Hospital, Dr. Molewaterplein 60, 3015 GJ Rotterdam, The Netherlands; Yulius Academy, Yulius, Organisation of Mental Health, Dordrecht, The Netherlands

**Keywords:** Autism spectrum disorder, Pervasive developmental disorders, Comorbidity, Follow-up

## Abstract

The current study was a 7-year follow-up of 74 6–12 year old children with Pervasive Developmental Disorder-Not Otherwise Specified. We examined the rates and 7 year stability of comorbid psychiatric diagnoses as ascertained with the Diagnostic Interview Schedule for Children: Parent version at ages 6–12 and again at ages 12–20. Also, we examined childhood factors that predicted the stability of comorbid psychiatric disorders. The rate of comorbid psychiatric disorders dropped significantly from childhood (81 %) to adolescence (61 %). Higher levels of parent reported stereotyped behaviors and reduced social interest in childhood significantly predicted the stability of psychiatric comorbidity. Re-evaluation of psychiatric comorbidity should be considered in clinical practice, since several individuals shifted in comorbid diagnoses.

## Introduction

Psychiatric comorbidities in children with autism spectrum disorder (ASD) are rather a rule than an exception (Abdallah et al. 2011; De Bruin et al. 2007; Ghaziuddin et al. 1998; Joshi et al. 2010; Leyfer et al. 2006; Simonoff et al. 2008, 2013; Van Steensel et al. 2013). In 2007, we showed that at least one comorbid psychiatric disorder was present in 81 % of the children with Pervasive Developmental Disorder-Not Otherwise Specified (PDD-NOS); 62 % had a comorbid disruptive behavior disorder, and 55 % fulfilled criteria of an anxiety disorder (De Bruin et al. 2007). Little is however known about the stability of these childhood psychiatric comorbidities in ASD towards adolescence and adulthood. Adolescence is marked by many social, psychological and physical changes in the individual. These changes are often accompanied by an increase in emotional or behavioral problems (Dahl 2004). The issues may be amplified in ASD as a result of their particular difficulties in adapting to change. Simonoff et al. (2013) have shown that comorbid psychiatric symptoms in ASD are persistent from childhood to adolescence, with domain-specific correlations (i.e. among one specific type of symptoms, such as anxiety) ranging from 0.33 to 0.58 across the period of 12–16 year old. However, the authors investigated the stability of continuous questionnaire measures rather than the stability of diagnostic categories. Information on the stability of diagnoses based on in-depth interviews with trained professionals can give us additional information on the impact of problems on everyday functioning.

Therefore, the current prospective study firstly investigated the stability from childhood into adolescence of comorbid DSM-IV defined psychiatric disorders in children with PDD-NOS on whom we previously reported childhood comorbidity rates (De Bruin et al. 2007).

It is important to know which individuals are at risk of persistent comorbid psychiatric disorders throughout adolescence. For this purpose, it is important to examine factors in childhood that may predict the persistence of comorbid disorders into adolescence. Several putative predictors can be derived from the literature. Individual factors, such as *age*, *gender*, *intelligence* and *type and/or severity of ASD symptoms*, might influence outcome in adolescence. Firstly, the variation in *age* of onset varies among comorbid disorders (e.g. Hepburn et al. 2014), thus, age might explain differences in outcome. Secondly, *gender* might also be an important predictive factor, since in the general population, gender differences of psychiatric disorders are often described. For example, anxiety symptoms in adolescence and depression in late adolescence are reported at higher prevalence in girls compared to boys (Van Oort et al. 2009; Costello et al. 2003; Petersen et al. 1991; Hankin et al. 1998). Disorders such as attention deficit/hyperactivity disorder (ADHD) and conduct disorder (CD) are described to be more often present in boys than in girls (Costello et al. 2003; Arnold 1996; APA 2000). Thirdly, *intelligence* might be a predictive factor since children in the general population with an Intelligence Quotient (IQ) below 80 tended to have more emotional and behavioral problems compared to children with an IQ above 80 (Dekker et al. 2002). In contrast, studies regarding children with ASD often report high rates of co-occurring disorders in individuals with both high and low IQs (Simonoff et al. 2008; Leyfer et al. 2006). Fourthly, *type and severity of ASD symptoms* might also influence the comorbid disorders. In our previous cross-sectional study we showed that children with PDD-NOS and both internalizing and externalizing disorders had more parent-rated problems with executive functioning (i.e. orientation in time, place and activity), more problems with tuning-into the social situation and more stereotyped behaviors than those with PDD-NOS without comorbid psychiatric disorders (De Bruin et al. 2007). Moreover, individuals with internalizing and externalizing disorders in childhood are at risk for developing other co-occuring disorders during adolescence (Goodwin et al. 2004; Bittner et al. 2007; Bussing et al. 2010). However, more studies are needed to evaluate the development of comorbid disorders in individuals with ASD. Therefore, in the current study, we also investigated whether gender, age, IQ, type and severity of parent-rated ASD symptoms, type of initial comorbid psychiatric disorder, intermediate mental health care and medication were associated with the stability of comorbid psychiatric disorders.

## Methods

### Sample and Procedure

Participants were 74 individuals with PDD-NOS, who were initially referred for diagnostic evaluation to the Department of Child and Adolescent Psychiatry/Psychology of the Erasmus Medical Center—Sophia Children’s Hospital between July 2002 and September 2004. Eighty eight percent of the sample was male, the mean IQ was 93 (*SD* = 16.96), 90.5 % had a Dutch nationality. 45 % of fathers and 46 % of mothers had had a post-secondary school education; 84 % of fathers and 57 % of mothers had had a paid job in the previous 2 years, and most were in paid employment at the time of this study (83 % of fathers and 54 % of mothers). Of 54 participants, their living situation was known; 87 % were living at home with their parent(s) at wave 2.

Inclusion criteria were: (1) meeting the research criteria for PDD-NOS in childhood (n = 94; De Bruin et al. 2007; Buitelaar et al. 1999), and (2) participation of the parents in the Diagnostic Interview Schedule for Children: Parent version ([DISC-IV-P], Ferdinand and Van der Ende 1998; Shaffer et al. 2000), in childhood (n = 94, wave 1; age 6–12, *M* = 9.02, *SD* = 1.81) and 7 years later, in adolescence (n = 74, wave 2; age 12–20, *M* = 16.00, *SD* = 1.92). The average follow-up time between the DISC-IV-P at wave 1 and wave 2 was 6.95 years (range 5.58–8.82 year; *SD*: 0.64).

The 74 individuals whose parents participated at both wave 1 and wave 2 did not significantly differ in terms of gender, age, nationality, parental nationality, socio-economic status and number of DISC-IV diagnoses from individuals whose parents participated only at wave 1 (n = 94) (*p* > .05). However, individuals whose parents participated at both wave 1 and wave 2 had significantly higher IQ-scores (*M* = 92.96, *SE* = 16.96) versus individuals whose parents only participated at wave 1 [*M* = 83.94, *SE* = 16.88; *t*(86) = −2.01, *p* = .05].

### Measures

#### Diagnostic Interview Schedule for Children IV Parent Version (DISC-IV-P)

The DISC-IV-P (Shaffer et al. 2000) is a structured parent interview determining 1- and 12-month DSM-IV-TR (APA 2000) psychiatric disorders in children and adolescents. Parents were interviewed by phone by trained and certified research assistants. The DISC-IV-P was used to assess internalizing disorders (i.e. anxiety and mood disorders) and externalizing disorders (i.e. disruptive disorders) at wave 1 and 2 and was scored using the internet software (Steenhuis et al. 2009) of the Dutch translation of the DISC-IV-P (Ferdinand and Van der Ende 1998). The anxiety disorder module consists of nine disorders; social phobia (SoPh), separation anxiety disorder (SAD), specific phobia (SP), panic disorder (PD), agoraphobia (AG), generalized anxiety disorder (GAD), selective mutism (SM), obsessive compulsive disorder (OCD) and posttraumatic stress disorder (PTSD). The mood disorder module consists of major depressive episode (MDD), dysthymia, and manic/hypomanic episode. The disruptive behaviors are subdivided in the DISC-IV-P in: ADHD, oppositional defiant disorder (ODD) and CD.

#### Children’s Social Behavior Questionnaire (CSBQ)

To examine whether the level and type of parent-rated ASD symptoms in childhood (i.e. wave 1) was associated with the stability of comorbidity, the CSBQ was used. The CSBQ is a parental questionnaire, which contains 49 items about a broad range of features that are typical for ASD (Hartman et al. 2006; Luteijn et al. 1998). The items are scored on a three-point scale (i.e. 0: behavior does not apply; 1: behavior sometimes or somewhat applies; 2: behavior clearly or often applies to the child). The CBSQ consists of six subscales; (1) “not optimally tuned to the social situation”, (2) “reduced contact and social interest”, (3) “orientation problems in time, place or activity”, (4) “difficulties in understanding social information”, (5) “stereotyped behavior” and (6) “fear of and resistance to changes”. Good test–retest, inter-rater reliability and internal consistency have been reported for this measure (Hartman et al. 2006). Across our sample, internal consistency of CBCQ data was good, with Cronbach’s alpha’s ranging from 0.79 to 0.88.

#### Intelligence Quotient (IQ)

To examine whether IQ in childhood (i.e. wave 1) was associated with the stability of comorbidity, the Wechsler Intelligence Scale for Children-Revised (WISC-R; Wechsler 1974) was administered. This instrument comprises a verbal scale (VIQ) and a performance scale (PIQ) together forming a total scale (TIQ). The Dutch version of the WISC-R has been demonstrated to be sufficiently reliable and valid (Van Haasen et al. 1986).

#### Intermediate Use of Mental Health Care and Medication

To assess the use of mental health care and medication between wave 1 and 2, a parent questionnaire was administered at wave 2 (Amone-P’Olak et al. 2010). Eight questions concerned use of mental health care with regard to emotional and/or behavioral problems of the child. These items were scored as 0 (i.e. not used) or 1 (i.e. used). If any of these items was scored 1, the variable ‘mental health care’ was scored 1. Parents were also asked if their child had used psychotropic medication in the past 2 weeks. This variable was also scored as 0 (i.e. not used) or 1 (i.e. used).

#### Self-Reported Emotional and Behavioral Problems (Youth Self Report; YSR)

The YSR is an instrument of the Achenbach System of Empirically Based Assessment (ASEBA) that has good reliability and validity (Achenbach and Rescorla 2001), and has been used previously with cognitively able individuals with ASD (Schroeder et al. 2011; Hurtig et al. 2009). Adolescents rate their behavior over the preceding 6 months, items being scored on a three-point scale, with responses: 0 = not true, 1 = somewhat or sometimes true, 2 = very true or often true. The problem items are scored on empirically based syndromes that were derived by factor analyses. In the current study, the YSR was only administered at wave 2, and we analyzed scores on the following scales: Anxious/Depressed, Withdrawn/Depressed, Somatic Complaints, Attention Problems, Rule-Breaking Behavior and Aggressive Behavior (i.e. the scales that are most similar to the content of the DISC-P). T-scores of 65 or higher reflect scores in the subclinical range, while t-scores of 70 or higher reflect scores in the clinical range.

#### Data Analysis

Firstly, the rates of comorbid psychiatric disorders were calculated at wave 1 and wave 2. The significance of putative changes in the prevalence rates was tested using McNemar tests. To investigate the stability of comorbid psychiatric disorders from childhood to adolescence, we made a cross-table of the presence or absence of comorbid disorders at wave 1 and wave 2 (i.e. proportions of individuals in the following groups: 1: “persistent presence”, 2: “from presence to absence”, 3: “from absence to presence” and 4: “persistent absence”). To graphically illustrate whether stability was domain-specific, the number of individuals with either continuous or discontinuous disorders from wave 1 to wave 2 were demonstrated.

To examine whether gender, age, IQ, level and type of parent-rated ASD symptoms (CSBQ scores), intermediate mental health care and medication were associated with the stability of psychiatric disorders, we compared the group with persistent disorders (n = 38, “persistent presence”) with the group that changed from disorder to no disorder (n = 22, “from presence to absence”) and the group with persistent absence of disorders (n = 7, “persistent absence”) with the group that changed from no disorder to disorder (n = 7, “from absence to presence”). Comparisons were performed using t-tests for continuous variables (i.e. IQ, age, CSBQ scores) and Chi Square tests for categorical variables (i.e. gender, mental health care use and the use of psychotropic medication). For the smaller groups “persistent absence” and “from absence to presence”, non-parametric testing (i.e. Mann–Whitney *U* test and Binomial test) was used.

To be able to further interpret the parent-reported DISC data in the light of self-reported emotional and behavioral problems in adolescence, we post hoc explored the proportions of individuals who scored in the subclinical (≥t65) and clinical (≥t70) range of the YSR at wave 2.

## Results

### Prevalence of Comorbid Psychiatric Disorders in Childhood and Adolescence

The prevalence of all separate comorbid disorders at wave 1 and wave 2 are presented in Table 1. Internalizing disorders significantly decreased over time; 60 % of the individuals in childhood met criteria for at least one internalizing disorder, in adolescence this rate dropped to 35 % [McNemar; *x*^*2*^ (n = 74) = 10.32, *p* = .001]. Note that especially the rate of anxiety disorders, significantly decreased from childhood (i.e. 55 %) to adolescence (i.e. 31 %) [McNemar; *x*^*2*^ (n = 74) = 10.32, *p* = .001]; the rate of mood disorders approximately stayed the same. The rates for externalizing (i.e. disruptive) disorders did not significantly change from childhood (i.e. 61 %) to adolescence (i.e. 51 %). Overall, the number of individuals who had a comorbid psychiatric disorder significantly changed from childhood to adolescence [McNemar; *x*^*2*^ (n = 74) = 6.76, *p* = .01]; during childhood, 81 % of the individuals had one or more comorbid psychiatric disorder, during adolescence at least one comorbid psychiatric disorder was present in 61 % of the individuals.

**Table 1:** Prevalence of comorbid disorders according to the DISC-IV-P (n = 74)

Disorder	Wave 1 (age 6–12) % of individuals	Wave 2 (age 12–20) % of individuals	*p*	Transitions in diagnosis		
				Stable^a^	Out of^b^	Into^c^
				n	n	n
*Anxiety disorders*	55.4	31.1	**0.001**	18	23	5
Social phobia	10.8	1.4	**0.02**	1	7	0
Separation anxiety disorder	6.8	4.1	0.69	1	4	2
Specific phobia	40.5	25.7	**0.04**	12	18	7
Agoraphobia	5.4	2.7	0.69	0	4	2
Panic disorder without agoraphobia	1.4	0.0	1.00	0	1	0
Panic disorder with agoraphobia	0.0	1.4	1.00	0	0	1
Generalized anxiety disorder	6.8	4.1	0.73	0	5	3
Selective mutism	0.0	0.0	–	0	0	0
Obsessive compulsive disorder	6.8	8.1	1.00	0	5	6
Posttraumatic stress disorder	0.0	1.4	1.00	0	0	1
*Mood disorders*	12.2	10.8	1.00	3	6	5
Major depression	8.1	10.8	0.73	3	3	5
Dysthymic disorder	2.7	0.0	0.50	0	2	0
Mania	4.1	1.4	0.63	1	3	1
Hypomania	2.7	0.0	0.50	0	2	0
*Disruptive disorders*	60.8	51.4	0.28	26	19	12
ADHD, combined type	20.3	8.1	0.05	2	13	4
ADHD, inattentive type	13.5	25.7	0.08	4	6	15
ADHD, hyperactive/impulsive type	10.8	5.4	0.39	0	8	4
Oppositional defiant disorder	35.1	27.0	0.29	12	14	8
Conduct disorder	9.5	2.7	0.18	0	7	2

Bold values are statistically significant (*p* < 0.05)

^a^Identical = Participants had the same diagnosis at T1 and at T2

^b^Out of = Participants did have the diagnosis at T1, but no longer at T2

^c^Into = Participants did not have the diagnosis at T1, but did have the diagnosis at T2

### Stability of Comorbid Psychiatric Disorders

**Fig. 1:**
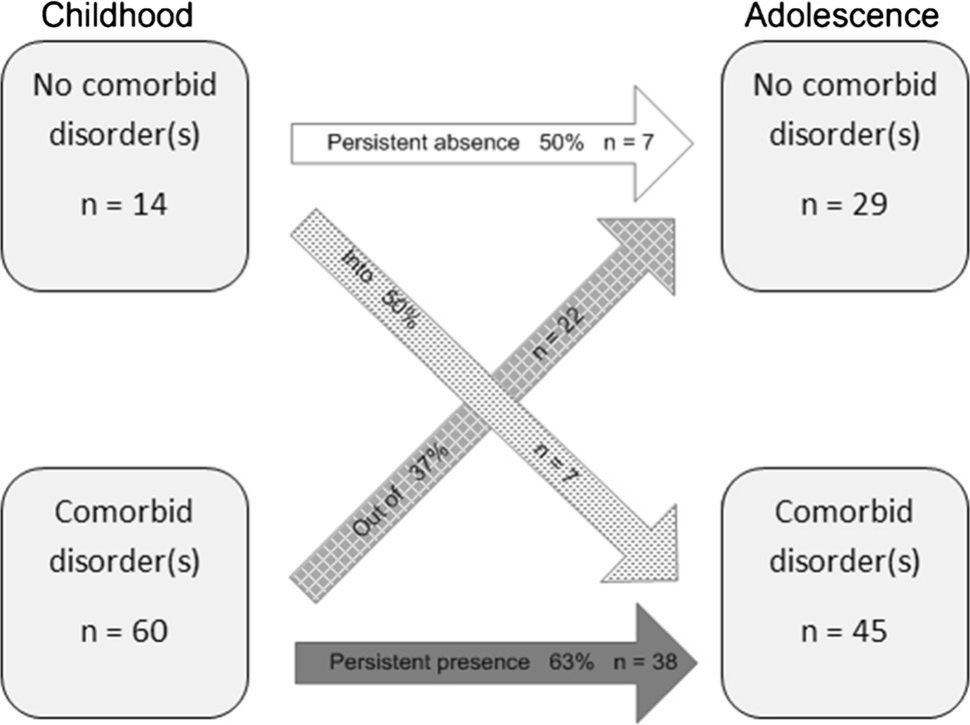
Stability of comorbid psychiatric disorders from childhood to adolescence

Figure 1 shows the proportions of individuals in the following groups (1) “persistent presence”, (2) “from presence to absence”, (3) “from absence to presence” and (4) “persistent absence”. Of the individuals who had at least one comorbid psychiatric disorder in childhood (n = 60), 63 % still had at least one comorbid psychiatric disorder in adolescence (n = 38), whereas 37 % of the individuals no longer met criteria for a comorbid psychiatric disorder in adolescence (n = 22). Of the individuals who had no comorbid psychiatric disorder in childhood (n = 14), 50 % (n = 7) stayed free of a comorbid psychiatric disorder in adolescence, whereas 50 % (n = 7) of the individuals developed at least one comorbid psychiatric disorder in adolescence.

**Fig. 2:**
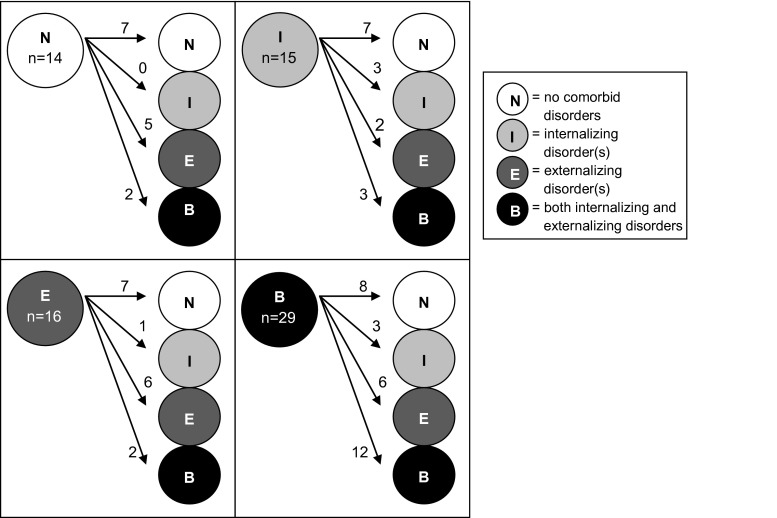
Transitions in type of comorbid psychiatric disorders from childhood to adolescence. This figure shows the different types of transitions from T1 comorbid diagnoses (*left circle in each square*) to T2 comorbid diagnoses (*right circles in each square*)

Figure 2 illustrates the domain-specific stability; the amount of individuals with continuous or discontinuous (i.e. other type of) disorders from wave 1 to wave 2.

### Predictors of Stability

The childhood characteristics of individuals in the “persistent presence” group (n = 38) versus the “presence to absence” group (n = 22) were compared to examine factors associated with stability of comorbid psychiatric disorders (Table 2). No significant differences were found regarding age, gender, IQ, intermediate mental health care and psychotropic medication.

**Table 2:** Childhood predictors compared between developmental course groups

	Persistent presence of psychiatric comorbidity (n = 38)	From presence to absence of psychiatric comorbidity (n = 22)	*p*	Persistent absence of psychiatric comorbidity (n = 7)	From absence to presence of psychiatric comorbidity (n = 7)	*p*
Gender: % boys	84 %	96 %	0.19	71 %	100 %	0.38
Age (SD)	9.06 (1.66)	8.94 (2.10)	0.83	9.29 (2.55)	9.71 (1.57)	1.00
IQ (SD)	91.65 (17.20)	97.79 (18.33)	0.22	92.57 (16.77)	87.14 (10.96)	0.54
CSBQ scores (SD)						
Not optimally tuned to social situation	13.76 (5.10)	12.24 (5.49)	0.29	9.00 (6.33)	8.57 (5.74)	0.90
Reduced contact and social interest	10.05 (5.55)	7.05 (4.54)	**0.04**	9.71 (5.77)	7.71 (3.45)	0.46
Orientation problems in time, place or activity	8.33 (3.73)	6.55 (4.64)	0.11	6.86 (3.76)	5.71 (1.89)	0.90
Difficulties in understanding social information	7.65 (4.07)	7.00 (3.81)	0.55	8.29 (3.50)	7.71 (3.15)	0.90
Stereotyped behavior	5.67 (3.90)	2.60 (2.37)	**0.001**	3.14 (4.63)	3.17 (2.86)	0.84
Fear of and resistance to changes	3.92 (2.03)	3.27 (2.41)	0.28	2.29 (1.98)	3.29 (1.89)	0.26
Mental health care	81 %	91 %	0.34	86 %	86 %	0.21
Medication	60 %	38 %	0.12	29 %	29 %	1.00

Bold values are statistically significant (*p* < 0.05)

As for level and type of symptoms of ASD, individuals with persistent comorbidity had significant more parent-reported stereotyped behaviors [*t*(53.465) = 3.66, *p* = .001, $R^{2}_{N}$ = 0.226] and reduced contact and social interest [*t*(57) = 2.15, *p* = .04, $R^{2}_{N}$ = 0.10] in childhood, than individuals who lost their comorbid psychiatric diagnosis during adolescence.

To further clarify this finding, a post hoc test was performed to reveal possible predictors specific for persistent externalizing versus persistent internalizing disorders. In both cases, only parent-reported stereotyped behavior was a significant predictive factor for persistence of the same psychiatric comorbidities [i.e. respectively (*t*(40) = −2.953, *p* = .005); (*t*(39) = −3.287, *p* = .002)].

The childhood characteristics of the “persistent absence” group (n = 7) were compared to the “absent to present” group (n = 7) on factors associated with stability of comorbid psychiatric disorders (Table 2). No significant differences were found in age, gender, IQ, level or type of ASD symptomology, intermediate mental health care or use of psychotropic medication.

### Self-Reported Emotional and Behavioral Problems

Table 3 shows the proportions of individuals who scored in the subclinical (t65–t70) and clinical (≥t70) range of the YSR at wave 2.

**Table 3:** Self-reported emotional and behavioral problems (n = 66) at wave 2

YSR scale	% (Sub)clinical range^a^
Anxious depressed	15.2 % (of which n = 6 subclinical, and n = 4 clinical)
Withdrawn depressed	18.2 % (of which n = 10 subclinical, and n = 2 clinical)
Somatic complaints	6.1 % (of which n = 0 subclinical, and n = 4 clinical)
Attention problems	18.2 % (of which n = 6 subclinical, and n = 6 clinical)
Rule breaking behaviors	4.5 % (of which n = 1 subclinical, and n = 2 clinical)
Aggressive behaviors	12.1 % (of which n = 5 subclinical, and n = 3 clinical)

^a^Subclinical range = t65–t70; clinical range ≥t70

## Discussion

The current follow-up study examined stability and predictors of comorbid psychiatric disorders in individuals with PDD-NOS. Firstly, we examined the rates of comorbid psychiatric disorders in childhood and in adolescence. The rates of individuals who had one or more comorbid psychiatric disorder in childhood (81 %) and adolescence (61 %) were similar to rates found in previous studies (Abdallah et al. 2011; Ghaziuddin et al. 1998; Leyfer et al. 2006; Simonoff et al. 2008; Van Steensel et al. 2013). In general, psychiatric comorbidity was rather stable (63 %); individuals who already had at least one comorbid psychiatric classification in childhood, usually still had at least one comorbid psychiatric classification in adolescence. One previous study concerning the stability of *symptom levels* of other psychiatric disorders from childhood to adolescence in a sample of individuals diagnosed with ASD found that symptoms of co-occurring psychiatric problems were persistent in individuals with ASD from childhood to adolescence (Simonoff et al. 2013). The current study added to that work by showing that both other psychiatric symptoms and other categorical psychiatric diagnoses can persist from childhood to adolescence. Changes in prevalence rates and transitions between types of comorbidity were examined and are discussed below. Due to the limited sample size, domain-specific stability of psychiatric comorbidity could not be tested.

The prevalence of one or more anxiety disorder in individuals with PDD-NOS, according to the DISC-IV-P, was significantly higher in childhood (55 %) than in adolescence (31 %). In the general population, also a decrease in anxiety from early to middle adolescence has been found (e.g. Van Oort et al. 2009), suggesting a similar developmental process. The rates for anxiety disorders in the individuals with PDD-NOS in the current study were quite high, although even higher rates (84 %) were reported in the literature (Muris et al. 1998). In the current study, among all anxiety disorders, the highest prevalence was found for SP at both assessment waves; 41 % in childhood, and 26 % in adolescence. This is in line with previous studies that also described SP as the most common anxiety disorder in children with ASD (Leyfer et al. 2006; Muris et al. 1998; Van Steensel et al. 2013) The current findings add to the existing literature, by showing that also in adolescence, SP is the most common anxiety disorder among individuals with ASD, although a significant drop was also found with 18 cases no longer meeting criteria for this disorder. Possibly, the finding of highly prevalent SP could be explained by the fact that we—and previous studies—used the parent as the informant; i.e. specific phobias might be most noticeable to parents, especially in childhood. For this reason, we also explored self-reported anxiety levels. These preliminary findings did not suggest higher self-reported levels of anxiety in adolescence. Yet clearly, further studies using self-reported anxiety are needed to clarify the most prevalent types of anxiety in children and adolescents with ASD (Muris et al. 1998).

The rate of MDD increased slightly from 8 % in childhood to 11 % in adolescence. These rates are in line with those known from the literature, although higher (i.e. 16 %) and much lower rates (i.e. 1 %) have also been reported among individuals with ASD (Leyfer et al. 2006; Simonoff et al. 2008; Mazefsky et al. 2011; Van Steensel et al. 2013; Witwer and Lecavalier 2010). In the general population, also an increase in depression from childhood to adolescence has been found (Maughan et al. 2013), suggesting a similar developmental process.

The rate of comorbid ADHD decreased from childhood (45 %) to adolescence (39 %). These relatively high rates are in line with previous studies (Goldstein and Schwebach 2004; Leyfer et al. 2006; Mazefsky et al. 2011), although lower rates have also been reported among individuals with ASD (Abdallah et al. 2011; Hanson et al. 2013; Simonoff et al. 2008; Van Steensel et al. 2013). The current study found a decrease for the hyperactivity type of ADHD and the combined type of ADHD in individuals with PDD-NOS from childhood to adolescence, but an increase for the inattentive type of ADHD in adolescence, with 15 new cases in adolescence. This change in the occurrence of ADHD in individuals with PDD-NOS is in line with the suggested trajectory of development of ADHD with hyperactivity at preschool age, more normoactive behavior during the early school years and a tendency to hypoactivity in early adolescence (Gillberg and Billstedt 2000). In adolescence, transition to secondary education increases attentional demands and peer pressures, with the potential to amplify previously unnoticed problems.

In the current study, criteria for ODD were met for 35 % of the individuals in childhood and 27 % of the individuals in adolescence. Simonoff et al. (2008) found similar rates (28 %) with the DISC in their study among 10–14 year old individuals with ASD, although much lower (i.e. 7 %; Leyfer et al. 2006) as well as higher (i.e. 75 %; Witwer and Lecavalier 2010) rates of ODD have been reported in studies that used other diagnostic instruments. Criteria for CD were met for 10 % of the individuals in childhood and 3 % of the individuals in adolescence. The rate in adolescence is similar to the rate (3 %) found in the study of Simonoff et al. (2008) among 10–14 year old individuals with ASD, although much higher rates (i.e. 22 %) are reported among 3–17 year olds with ASD (Joshi et al. 2010). All individuals with CD in the current study also met the criteria for ODD at both T1 and T2.

Taken together, the overall prevalence of parent-reported psychiatric comorbidity decreased from childhood to adolescence. Variation in the prevalence of comorbid disorders across different studies may be explained by methodological differences in aspects such as methods of assessment and diagnosis, and how samples were constituted (i.e. differences in levels and types of care in different institutions). Our own data showed no particular transitions from one type of comorbidity to another; only 2 cases shifted from having an internalizing to an externalizing diagnosis, and one from an externalizing to an internalizing one. Although our dataset is limited, cross-domain transitions seem infrequent, and domain stability seems most common.

The current study suggested two predictors of stable psychiatric comorbidity from childhood to adolescence. Children with stable psychiatric comorbidity showed a higher childhood level of parent-reported stereotyped behavior and reduced contact and social interest. Post-hoc analyses also showed that parent-reported stereotyped behaviors were predictive for the persistent presence of either internalizing or externalizing disorders. Thus, our preliminary findings suggest that parent-reported stereotyped behaviors might be an indication for persistent comorbidity. There were no significant predictors for the persistent absence of comorbidity, although there probably was a lack of power to detect these, since these groups were very small. To our knowledge, no previous studies investigated childhood predictors of comorbidity in adolescence, so further research is needed to corroborate our findings.

Taken together, although the stability rate of any comorbid psychiatric classification was considerable (63 %), re-evaluation of psychiatric comorbidity in adolescence should be considered in clinical practice, since a reasonable amount of individuals no longer meet diagnostic criteria for a comorbid classification in adolescence or shifted from one comorbid diagnosis to another. Reduced social interest and stereotyped behavior as rated by parents might be considered as indicators of stable psychiatric comorbidity.

PDD-NOS itself can show different developmental trajectories. In the current literature, there are studies that show relative stability of ASD diagnosis (Jónsdóttir et al. 2007; Takeda et al. 2005); but also studies showing shifts within the spectrum and studies showing that some individuals appear to move out of the spectrum (Daniels et al. 2011; Kleinman et al. 2008; Turner and Stone 2007; for a review, please see Woolfenden et al. 2012). Of the 74 children with PDD-NOS participating in this last study, 22 no longer met criteria for PDD-NOS in adolescence. There are also children that don’t meet criteria for ASD at a young age, but who do at an older age (i.e. in our larger cohort of 246 clinical referrals, 16 children did not meet PDD-NOS criteria in childhood, but did so in adolescence). Changes in comorbidity need to be interpreted in the context of these possible changes within ASD. For details on the stability of ASD within this sample, see Louwerse et al. (2015). Further research into the intertwined development of ASD symptomology and autistic and co-occurring psychiatric problems is warranted.

Our sample of children was diagnosed with PDD-NOS. But one may wonder how these findings apply to individuals diagnosed with ASD using current DSM 5 criteria. The children in our sample also were part of a larger study on phenotypic profiles of children with PDD (Greaves-Lord et al. 2013). This study has found that about 30 % of the sample had a profile more in line with the more recent DSM-5 diagnosis of Social (Pragmatic) Communication Disorder. The reader should interpret our findings against this background.

Our wave 2 YSR self-report data was used to provide information on adolescents’ internalizing comorbidities through post hoc exploration. Contrary to our expectations we found lower self-reported rates of anxiety rates (15.2 %) than parent-reported DISC-P wave 2 rates (31.1 %). We had expected to see higher self-reported than parent-reported rates of internalizing problems, given that adolescents might not openly share these emotions with their parents. In line with expectations, we found a somewhat higher rate of self-reported depressive symptoms (18.2 %—in the subclinical range) when compared to the parent-reported DISC-P wave 2 rate of 10.8 %. DISC-P data cannot be directly compared to YSR data due to the rather different properties of these instruments, and the lack of comparative research on these two measures. We hope these data provide some background information that helps to broaden the picture from our main analyses. Further research would be needed to profile the longitudinal trajectories of self-reported comorbidities in this group.

As discussed above, the current findings presented are quite preliminary, and should be interpreted against the background of some methodological limitations. Results are based on parental interviews in a relatively small sample of individuals with PDD-NOS with an average to high IQ who were referred to one particular center, thus clinicians should make careful considerations regarding their own specific clients, and further research on samples with more phenotypic variation in ASD severity and cognitive ability using multiple informants is needed. Also, only information on current medication in adolescence was obtained, without further details on medication history. Therefore, our finding that medication was not associated with later comorbidity should be further scrutinized in future research. Finally, since only 12 participants of the current sample were 18 years or older at the time of wave 2 data collection, it is not yet possible to shed further light on the adult outcomes of the current sample. Development from adolescence into adulthood is accompanied by many challenges regarding societal functioning (i.e. community and social participation, such as obtaining a job, living independently, and building intimate relationships). In a study of Myers et al. (2015), it was found that co-occurring behavioral difficulties in adolescents with ASD were associated with increased risk of social isolation in adolescence, but not with social or community participation in adulthood. This study did not investigate behavioral or emotional difficulties in adulthood, but one might assume that comorbid emotional and/or behavioral problems in adulthood may be affected by negative social/community outcomes, and may thus become more problematic over time. Future research is warranted to further examine the trajectories and determinants of comorbid psychiatric disorders of individuals with ASD into adulthood and later life.
